# Experiences of Gamified and Automated Virtual Reality Exposure Therapy for Spider Phobia: Qualitative Study

**DOI:** 10.2196/17807

**Published:** 2020-04-29

**Authors:** Philip Lindner, Alexander Rozental, Alice Jurell, Lena Reuterskiöld, Gerhard Andersson, William Hamilton, Alexander Miloff, Per Carlbring

**Affiliations:** 1 Department of Psychology Stockholm University Stockholm Sweden; 2 Center for Psychiatry Research Department of Clinical Neuroscience Karolinska Institutet & Stockholm Health Care Services, Region Stockholm Stockholm Sweden; 3 Department of Behavioral Sciences and Learning Linköping University Linköping Sweden; 4 Mimerse Stockholm Sweden

**Keywords:** virtual reality, gamification, serious game, exposure therapy, phobia, user experience

## Abstract

**Background:**

Virtual reality exposure therapy is an efficacious treatment of anxiety disorders, and recent research suggests that such treatments can be automated, relying on gamification elements instead of a real-life therapist directing treatment. Such automated, gamified treatments could be disseminated without restrictions, helping to close the treatment gap for anxiety disorders. Despite initial findings suggesting high efficacy, very is little is known about how users experience this type of intervention.

**Objective:**

The aim of this study was to examine user experiences of automated, gamified virtual reality exposure therapy using in-depth qualitative methods.

**Methods:**

Seven participants were recruited from a parallel clinical trial comparing automated, gamified virtual reality exposure therapy for spider phobia against an in vivo exposure equivalent. Participants received the same virtual reality treatment as in the trial and completed a semistructured interview afterward. The transcribed material was analyzed using thematic analysis.

**Results:**

Many of the uncovered themes pertained directly or indirectly to a sense of presence in the virtual environment, both positive and negative. The automated format was perceived as natural and the gamification elements appear to have been successful in framing the experience not as psychotherapy devoid of a therapist but rather as a serious game with a psychotherapeutic goal.

**Conclusions:**

Automated, gamified virtual reality exposure therapy appears to be an appealing treatment modality and to work by the intended mechanisms. Findings from the current study may guide the next generation of interventions and inform dissemination efforts and future qualitative research into user experiences.

## Introduction

Virtual reality (VR) refers to technology that creates an immersive experience of being present in a virtual, computer-generated world. Today, this is typically achieved through the use of a head-mounted display (HMD) with stereoscopic screens that withhold the outside world and allow the user to look around the virtual world by measuring head rotation and adapting the visual presentation accordingly [1]. Until recently, VR was an expensive, inaccessible, cumbersome technology with poor graphical quality that required specialized skills to operate [2], yet innovative clinical applications emerged nevertheless in the 1990s [3]. Over a dozen high-quality clinical trials have since supported the efficacy of VR exposure therapy (VRET) for the treatment of anxiety disorders [4-6], in which phobic stimuli are replaced with virtual equivalents to perform otherwise typical exposure therapy (ie, graded, systematic exposure under controlled conditions until the fear response habituates or inhibitory learning occurs) [7]. Importantly, this treatment works across age groups [8], the fear reduction in VR generalizes to real-world equivalent stimuli [9], there are low rates of deterioration [10], and clinicians have positive attitudes toward using the technology for therapeutic purposes [11,12].

The recent advent of consumer VR technology presents a paradigm shift in the design and dissemination potential of VRET interventions [13]. In particular, recent research has explored whether this type of intervention can be automated (ie, delivered without any real-life therapist and relying instead on gamification elements). Gamification is commonly defined as the application of traditional game elements, originally designed for enjoyment, to other contexts [14]. Common gamification elements include tasks relying on game mechanics, and reinforcement of progress and achievements through points and badges. When used for an explicit primary purpose other than enjoyment, the term serious game applies [15]. A coherent combination of gamification elements such as onboarding, level design, narrative building, performance feedback, and avatar assistance is inherently well suited to replace many of the tasks otherwise performed by a real-life therapist, meaning that a complete, self-contained serious game can be designed to be played and completed without a therapist directing the intervention, while still containing evidence-based treatment components [16]. To our knowledge, only three randomized clinical trials of automated VRET interventions have been published to date: two for fear of heights [17,18] and one for spider phobia [19]. Although all three studies reported large effect sizes in terms of symptom reduction, none reported on any user experiences beyond providing quotes from four participants on treatment effects [17], system usability ratings and cybersickness scores [18], and common negative effects [19]. A recent single-subject replication trial of the same VRET intervention for spider phobia reported descriptive statistics on app engagement and use, and additionally found no associations (eg, between cybersickness scores and intervention outcomes), although these correlational analyses had low power [20].

Automated, gamified VRET apps constitute a novel merger of gaming and technology with classic psychotherapy, and user experiences will likely be shaped by both technology-specific and therapeutic aspects. Design considerations for such interventions should be informed by a clear therapeutic rationale and preclinical research [13], while also taking user experiences into account so that the interventions are not only efficacious but also appealing. This is particularly relevant for consumer-targeted VRET apps intended for release on ordinary digital marketplaces, which will realistically compete for user time and interest with other apps (eg, pure games). As an example, a first-generation consumer-targeted VR relaxation app had a low degree of recurrent users despite being downloaded over 40,000 times (even in the infancy of consumer VR) and enjoying high ratings on the digital marketplace [21]. This reveals that although the intervention was well-received by a subset of users, most users did not find it appealing enough to use it multiple times. Careful mapping of user experiences may inform design decisions that avoid such outcomes.

Notwithstanding the demonstrated efficacy of VRET and other types of VR interventions for health, both in the automated and traditional format, very little is known about how users experience these interventions. Qualitative research on nongamified VRET has uncovered themes pertaining to the user’s sense of presence in the virtual environment, along with factors that break presence [22]. Congruently, previous qualitative research in adjacent fields has revealed that VR allows users to *experience* rather simply perceive different situations [23], and that VR technology has an appealing allure in itself, being exciting, novel, and enjoyable through the inherent feature of creating a sense of presence [24]. To attain a first glimpse into user experiences of undergoing automated, gamified VRET to guide the development of future iterations and quantitative research, we conducted a pilot explorative qualitative interview study in parallel with a randomized controlled noninferiority trial comparing this novel treatment approach to traditional in vivo exposure therapy for spider phobia [19,25].

## Methods

### Trial Design, Participants, and Procedure

The clinical trial (2015/472-31) and the parallel interview study (2015/1695-32) were approved by the Stockholm Regional Ethical Review Board.

Participants recruited for the interview study had completed the screening battery for the parallel clinical trial [19,25] but were not among the first 100 participants to be scheduled for and complete the on-site pretreatment assessment. Once the clinical trial had reached its recruitment goal, remaining participants were invited to join a reserve list for future similar studies. Ten participants expressed interest in joining a qualitative study entailing the same assessment procedure (before and after treatment), receiving VRET, and completing a face-to-face interview in conjunction with the posttreatment assessment. Among this group, one participant dropped out before the pretreatment assessment, one participant dropped out before the treatment session, and one participant dropped out before the posttreatment assessment, leaving a final sample of 7 participants. 

Sample size considerations for qualitative studies is an ongoing topic of debate with little consensus across fields and great variation in the extant literature [26]. The sample size for the current study was upper-bounded by the availability of participants as determined by the recruitment to the parallel clinical trial, and generalization was a priority. The final sample size was deemed to be acceptable owing to the pilot and explorative nature of the study, with an explicit aim of informing subsequent research and a relatively homogenous sample undergoing a fully standardized intervention. Given the former consideration, a lower number of theme instances was deemed to be sufficient, and given the latter considerations, a high sample theme prevalence was to be expected. This meant that the final sample size of 7 was within the realm of acceptability according to guidelines [27], in addition to being in accordance with the minimum acceptable sample size suggested by meta-analyses of published qualitative studies in the field of psychology [26]. The final sample comprised 86% women with a mean age of 36.29 (SD 13.38) years, similar to the demographics of the VRET arm in the clinical trial [19].

Participants completed the same assessment procedure and received the same VRET intervention as in the clinical trial. All three parts took place on site at Stockholm University. Approximately 1 week prior to treatment, participants completed the pretreatment assessment consisting of a standardized behavioral approach test (BAT) [28], a diagnostic interview, and self-rating scales. The BAT featured a real-life, medium-sized spider (approximately 2 cm in diameter including the legs); only harmless varieties common to Sweden (such as *Tegenaria* and Araneidae) were used. The stated goal of the BAT was to enter a room, approach the spider contained in a transparent plastic container, pick it up, and hold it for 20 seconds. Participants were rated on a standardized scale (0-12) depending on how close they came to achieving this objective (see [19] and [29] for more details). The self-rating scale battery included the Fear of Spiders Questionnaire (FSQ) [30], which is an 18-item scale covering different spider phobia symptoms based on a 7-point response format with a theoretical score range of 18-126.

Treatment took place in a single 3-hour session (see below). Approximately 1 week later, participants completed the combined interview and posttreatment assessment (BAT and self-rating scales). Although the exact durations of interviews were not recorded, 15-30 minutes was allocated to the interview part of the posttreatment assessment. All interviews were conducted in Swedish (all participants spoke fluent Swedish, as per the inclusion criteria of the parallel trial) according to a semistructured interview guide comprising the following seven topics: treatment expectations, use of hardware and software, the virtual environment, the virtual therapist and spider expert, gamification elements, exposure elements, and satisfaction and progress. The audio-recorded interviews were conducted and transcribed verbatim by one author (AJ) who is a native Swedish speaker.

**Figure 1:**
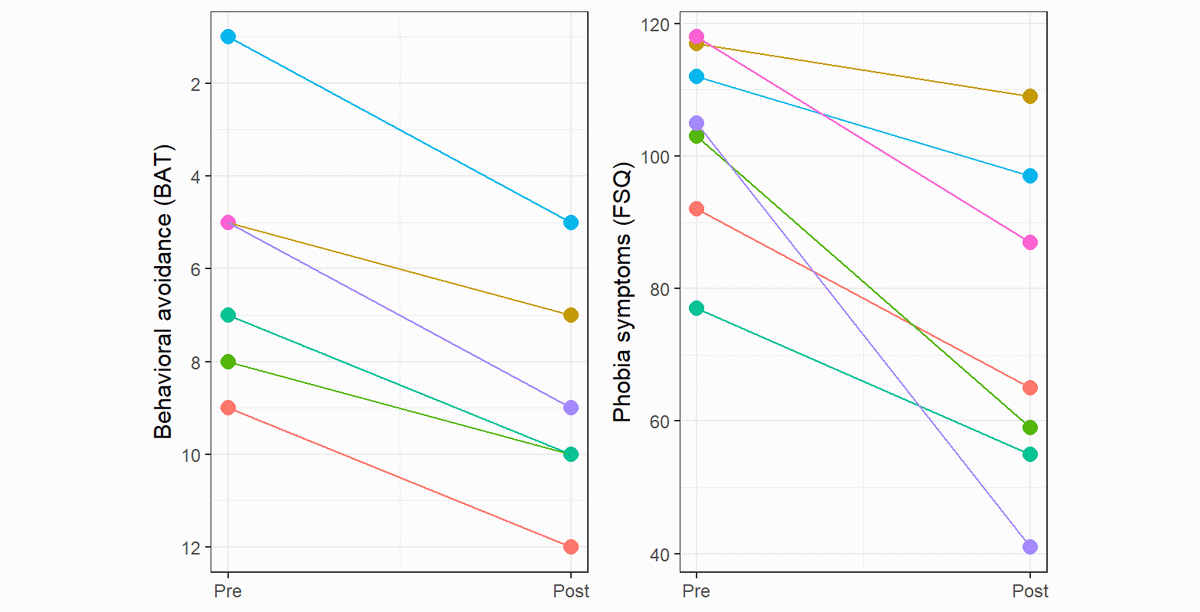
Spaghetti plots of individual participant treatment outcomes pre and post treatment. Different colors denote different individuals. Left panel: scores on the behavioral approach test (BAT) with a real spider (a higher score indicates less avoidance). The Y-axis is flipped for consistency. Right panel: scores on self-rated Fear of Spiders Questionnaire (FSQ) measuring the fear of spider symptoms (a lower score indicates fewer symptoms).

Participants achieved significantly higher BAT scores (reduced avoidance, *t_5_*=–8.22, *P*<.001) and reported significantly lower FSQ scores posttreatment (*t_6_*=4.23, *P*=.006), similar to the results of the VRET arm in the clinical trial. Table 1 compares the pre and posttest values in the BAT and FSQ between the present study and clinical trial, and Figure 1 shows a spaghetti plot of the changes for individual participants.

**Table 1:** Treatment outcomes in the present study and clinical triala.

Time	Behavioral Approach Test, mean (SD)		Fear of Spiders Questionnaire, mean (SD)	
	Qualitative study	Clinical trial	Qualitative study	Clinical trial
Pretest	5.71 (2.63)	4.76 (2.71)	103.43 (14.73)	95.82 (15.26)
Posttest	8.83 (2.48)	8.50 (2.29)	73.29 (24.75)	70.35 (22.45)

^a^Data are from participants in the virtual reality exposure therapy arm in the clinical trial [19].

### Treatment

**Figure 2:**
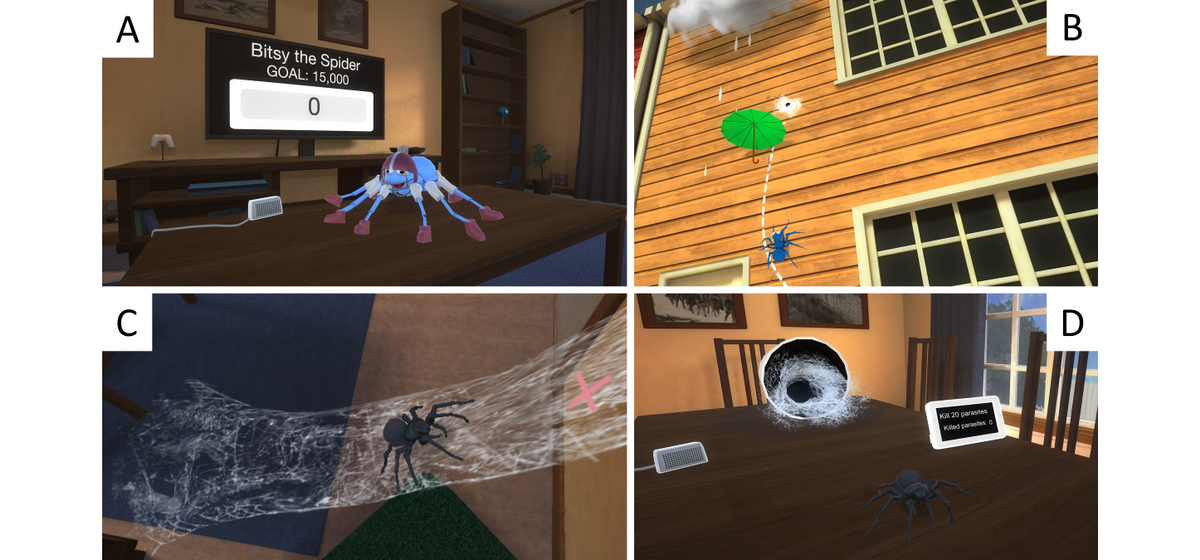
Screenshots from the virtual reality exposure therapy (VRET) app. (A) Example of a spider featured in an early, gaze-based level. (B) Example of a gamified exposure task (preventing the spider from being washed off the wall by moving the umbrella using gaze focused on the moving spider). (C) Example of realistic spider in a subsequent, boss-type level. (D) Example of a realistic spider used in higher levels.

The Itsy app is an automated VRET intervention designed as a serious game around classical exposure therapy elements [31]. The intervention starts with a psychoeducational introduction through a voiceover virtual therapist that conveys a cognitive behavioral conceptualization of spider phobia and provides a treatment rationale for exposure therapy. This psychoeducation takes place in a virtual therapist office with a projector for display purposes [13]. Once completed, the user progresses through 8 levels of exposure tasks, each with 3 sublevels: a simple gaze task (keep focusing on the moving spider to gain points), a game (eg, keep a moving spider safe by stopping objects from hitting it), and “boss” level (keep focusing on approaching the spider or it will turn away). The spider stimuli are increasingly realistic and frightening when progressing across the levels, beginning with a cartoon-looking, smiling spider, and ending with a realistic Black Widow spider. Spider movement animations were designed to be realistic and dynamic (ie, interactive to user behavior and not scripted). All sublevels featured gamification elements along with an overarching gamified structure. Table 2 summarizes the gamification aspects grouped according to a definitional framework derived from a systematic review of the extant gamification literature [14]. At the beginning and end of each sublevel, the user was prompted to rate subjective units of distress using a 0-100 scale. See Figure 2 for representative app screenshots. In addition to the virtual therapist, a voiceover spider expert was also included who presented facts about spiders (eg, descriptions of different spider species along with their biology and role in the ecosystem).

**Table 2:** Gamification elements included in the intervention.

Gamification element	Description
Dual-purpose game mechanics	All games designed to be both enjoyable and therapeutic, requiring the user to keep their gaze on a moving spider, with or without additional game mechanics. No included first-person movement, to both evoke common fear of invasion of private space by the spider and to avoid cybersickness.
Speed	Moving spider stimuli to evoke a greater fear response and prevent virtual reality-specific safety behavior of closing one’s eyes.
Goals	Clear goals for completion of each sublevel, conveyed verbally or visually.
Performance feedback	Scores displayed at all times and users could replay levels to achieve a higher score.
Badges and achievements	Visual summary of levels completed.
Dual-purpose narrative	Many sublevel games presented with a short narrative on task background and goal.
Points	Scoring key to game mechanic in gaze task and “boss” type sublevels, requiring a certain score to complete.
Levels	Familiar level design with levels and sublevels.
Increasing difficulty	Increasing spider realism with each level.
Onboarding/psychoeducation	First part of game features traditional cognitive behavioral therapy psychoeducation on phobia development and maintenance, and rationale for exposure.
Virtual helper	Voiceover virtual therapist, also presented as hologram avatar in the virtual therapist room, introduced at beginning of game and guiding the user throughout, giving instructions, encouraging progress and achievements, and summarizing key therapeutic points.

The Itsy app was designed for and operated on a Samsung (Seoul, Republic of Korea) smartphone (Note 4 or Galaxy S6) together with a first-generation Samsung Gear VR headset (ie, mobile VR) and inexpensive headphones. At the time, no hand controllers were available for the Gear VR platform; users interacted with the virtual environment via the headset touchpad and a gaze-directed crosshair. Users could pause the app at any time using a physical button on the headset. While automated, treatment was delivered with a “technician” in the room to assist in case of technical difficulties or a severe emotional reaction, as in the parallel noninferiority trial. At the time of data collection, the occurrence of severe emotional reactions was unknown and although not systematically recorded in the current study, only 10% of VRET participants in the parallel clinical trial were provided with qualified support requiring at least some expertise in clinical psychology (eg, discussing and coping with catastrophic beliefs) [19]. The duration of app use and rates of full completion were not systematically recorded in the current study; however, in the subsequent replication trial, all but one participant (96%) completed the full intervention in the same allocated time as in the current study.

### Qualitative Analysis

Inductive thematic analysis was used to explore the transcribed interviews in accordance with established guidelines [32]. This method was selected since no tailored theoretical framework exists concerning users’ experiences of undergoing automated, gamified VRET for specific phobia. The material was analyzed by one author (AR), who is a native Swedish speaker and a clinical psychologist and researcher with extensive experience of cognitive behavior therapy, internet-based psychological treatments, and qualitative studies, but not of the VR field. AR was not involved in the parallel clinical trial from which the participants were recruited, nor has any financial or other interests in the equipment or software used. No dedicated software for thematic analysis was used.

AR first familiarized himself with the material by repeatedly reading and exploring its content, followed by coding recurrent ideas put forward by the participants. In this case, the coding process can be considered selective [32], as the main purpose of the current study was to explore the expectations, experiences, and outcomes of participants undergoing treatment. The codes can be considered the building block of the analysis: examples such as “feeling unreal,” “animated,” “pretending,” and “no tactile stimulation” reflected the semantic or explicit meaning of the material as no interpretative framework was being used. The codes were then examined consecutively to find possible themes and subthemes, which were further reviewed and refined by revisiting and reexamining the material numerous times, such as “Expectations,” “Doubts,” and “Lack of expectations.”

## Results

### Thematic Structure

The inductive thematic analysis resulted in 7 themes and 8 subthemes, which are presented in Table 3, along with the covered codes. Representative quotes on each theme are provided below. The quotes were translated into English from the original Swedish by the researcher (AR) that conducted the thematic analysis; validity of these translations was assessed by independent backtranslation into Swedish by another author (PL) and differences were resolved collectively.

**Table 3:** Themes and subthemes from qualitative analysis.

Themes and subthemes		Codes
**Expectations**		
	Doubtful	Feeling unreal, cartoonish, make-believe, no tactile stimulation, simulated, unreasonable, short duration, too simple, level of fear, duration of phobia, routines, being different, lack of expectations, avoidance.
	Hopeful	Feeling safe, easier than the real thing, less afraid, research study, cognitive behavior therapy, providing some relief.
Becoming absorbed		Surprised, engaged, better than expected, forgetting reality, forgetting it is treatment, real movements, realistic, well-done, anxious, scared, crying, scary, reacting, trying to hide, trying to avoid, being focused.
Good enough		Safer than the real thing, good that not too realistic, control.
Simplicity		Thought-through, sympathetic/empathetic, novice, pedagogical, focus, undramatic, gamification, tasks, increasingly more difficult, interaction, generational issue, being a gamer.
**Psychoeducation**		
	Understanding your fears	Calming voice, confronting your fear, subjective units of discomfort, informative, normalizing, fears, expert, assumptions, like school, learning about phobias.
	Getting worse	Trying to avoid, negative association, increasing your fear.
**Problems and glitches**		
	Technical issues	Visual perspectives, acuity, lack of details, menus, not real enough, getting stuck, pauses, wrong sequence, stopped working, overheated, battery issues, clumsy, computer errors, restricted movements.
	Human issues	Becoming nauseous, difficult exercises, difficult understanding, panicky, time constraints, frustration, contacts and glasses, overwhelmed, without notice, without warning.
**Outcomes**		
	Benefits	Less anxious, less attentive to fears, continued exposure, proud, applications in real life, less worried, approaching spiders, improved, recommending treatment to others, increased knowledge, confident, quick response, not the whole world, seeing things differently, daring, significant others.
	Obstacles	Expensive equipment, more information needed, more exercise needed, doing it again, still scared, wrong season.

#### Expectations

Many participants were doubtful that automated VRET would actually work for their specific phobia and expressed concerns that the treatment did not involve real spiders or any sensory stimuli except for visual representations. Ideas about VR feeling too unreal and cartoonish were also mentioned, and that the treatment might be perceived as make-believe or simulated. Others conveyed skepticism toward what was seen as an intervention that was overly simplistic or too short in duration for something they had been struggling with for years and had become part of their daily routines. Some participants were slightly more optimistic, but believed their fear to be different or too intense in order for VRET to work; others lacked expectations altogether or tried not to think about it so that they would not become fearful.

I think I had some, I doubted how it would work since this was something that you know, I’ve only tried VR a couple of times before and I didn’t know how realistic it would feel.

Participant #1

Apart from the participants who had some doubts about whether VR would work for them, others were much more positive toward the treatment or believed it would be effective, despite some initial doubts. These participants highlighted the fact that a virtual environment would feel safer than engaging with spiders in real life, making it easier to take the steps necessary to manage their fears, or at least providing some relief. Others pointed out that they were part of a research study that was based on cognitive behavior therapy, which made them more confident that the VR intervention could work.

I had quite high expectations, since it was research and because it involved cognitive therapy which I’ve got great experiences of. So they were quite high.

Participant #4

#### Becoming Absorbed

Once engaged with treatment, most of the participants were surprised by their experiences of the virtual environment. They expressed being absorbed, that it exceeded their expectations, and that they almost forgot that they were seeing things through an HMD. More specifically, several participants described how real the spiders felt to them, especially how the movements of their legs were accurate and made them scared and anxious. A few participants even talked about jumping out of the chair, trying to hide, or crying, while others had a hard time letting the spiders out of their sight because of their fear.

Ehm, it wasn’t real spiders, you know, but at the same time, it felt very scary because they, it’s similar to how they behave in real life.

Participant #7

#### Good Enough

Not all participants were astonished by the virtual environment, complaining about the graphics and stating that it did not seem real. However, they still contended that it worked and was “good enough.” Moreover, a few participants said that it was better to use something that is a bit flawed and “sketchy” as it felt safer than exposing themselves to the real object of their fear. One participant even mentioned that it helped her feel in control, thereby allowing her to go through with all of the steps in treatment.

It wasn’t bad, it was okay, you know. I mean, it could be improved. But it was, basically, good enough for its purpose.

Participant #3

#### Simplicity

One important aspect raised by many of the participants was that the treatment felt simple, well thought out, and pedagogical. In particular, the outline was referred to as easy to grasp even for someone who had no prior experience of cognitive behavior therapy or had never previously used VR. In particular, some participants commented on the tasks in the game becoming increasingly more difficult, that it felt undramatic, and that it even made you feel sympathetic or empathetic toward the spiders. Some liked the fact that the treatment was framed as a game, making you more willing to focus and interact with the virtual environment. One participant noted that this type of outline could be particularly attractive to a generation of gamers who are accustomed to video and computer games.

Ehm, I thought it was nice except for when things crashed, but it was simple enough so that you would not just focus on the game but on the interaction with the spider, you know. Yeah, I thought it was great, you know, they increased the levels great, you know.

Participant #1

#### Psychoeducation

One feature of the treatment involved receiving psychoeducation from a voiceover therapist about specific phobia and managing anxiety through exposure. A male voice acted as a virtual therapist while a female voice acted as an expert who provided more general information about spiders. Some participants said that they enjoyed this support as it made them understand their fears better. In particular, they emphasized the calming nature of the virtual therapist and that the psychoeducation was informative and normalizing. A few participants mentioned that they liked exploring their beliefs about spiders and testing them out in VR, while others felt helped by labeling their anxiety level using subjective units of discomfort and rating whether their anxiety level increased or decreased during treatment, both of which were instructions provided from the voiceover therapist. One participant described the support as similar to being in school again, but learning about your worst fears and how to deal with them.

Yeah, and you also got to know a lot about spiders, that was really good, nice and calming voice that talked about spiders. That felt really great. So she could have talked some more.

Participant #2

However, not all participants experienced the voiceover therapist in the same way. Some felt annoyed by the voices and tried to focus on what was in front of them instead. Others started experiencing the female voice (the spider expert) negatively as the information she provided covered topics they were fearful of. To some extent, hearing about their worst fears made the anxiety worse.

No, for the most part I didn’t listen, I wasn’t interested in knowing, and then during level six, I think, I just felt that it was annoying that she kept going on, because I was really afraid, and got even more afraid when she started talking about 3000 babies, and I thought, I don’t want to listen to that to be honest. It all just became too much.

Participant #5

#### Problems and Glitches

Some problems were encountered during treatment, with several participants complaining about technical issues that either interfered with their engagement in the virtual environment or put everything to a halt. Some of these issues were related to the milieu they were in, such as visual perspectives or the visual acuity being off-putting, or that the details were all wrong and did not feel real enough. Others complained about menus in the game not working, that they got stuck during one phase or another, or that the sequence of tasks did not seem correct, such as when one level induced less fear than those they had already passed. Most participants also brought up the fact that the HMD stopped working completely, overheated, had battery problems, or that the software crashed altogether, which paused treatment for some time. Moreover, a few participants were frustrated by the equipment, which they described as being clumsy and restricting their movements.

Yes, that’s the thing, I had a stroke of bad luck, it crashed on me and the therapist said that this hadn’t happened before, not when he’d been using it, yeah, it was a bit problematic, it asked for some battery and then it just, it stopped and froze sometimes.

Participant #4

However, other difficulties were related to human issues, or that certain aspects of the treatment created problems for some participants. One of the most recurrent complaints was related to becoming nauseous after being in the virtual environment for an extended period of time, which meant that it was necessary to take a break. Others brought up aspects such as not being able to see things clearly because they were unable to wear their glasses in the HMD or that their contact lenses interfered with specific visual elements. A few participants also felt overwhelmed and panicky by some components in treatment, such as when spiders emerged in the game without notice or warnings. In addition, several participants had trouble understanding certain parts of the treatment, including how to rate your anxiety level or what to do in some levels of the game. One participant also complained about the length of the treatment, referring to time constraints in her personal life and feeling frustrated about the duration of several exercises she needed to complete in order to pass to the next level:

No, I just felt stupid when I was supposed to help one of the spiders from being hit by a ball, I sat there for ages, I was just like, I don’t understand!

Participant #6

#### Outcomes

Overall, participants were able to describe a number of benefits from undergoing the treatment, ranging from being less anxious, worried, and attentive to their fears to continuing applying the lessons they had learned such as using exposure and to approach spiders in everyday life. These participants described themselves as improved and more confident about managing difficult situations, as well as knowing more about what their fears were and how to deal with an episode of anxiety. Some participants also talked about seeing things differently, such as when referring to their own responses as normal and that it would not be the end of the world if they became anxious when seeing a spider. A few participants even brought up the fact that their significant others such as a partner had noticed a change and were praising them for being more daring in situations they previously would have avoided altogether.

I believe it’s better, I’m still no friend of spiders, but it’s like when I went over to visit my sister recently, there was a spider there, which I looked at, but it was dead and just lied there. I didn’t remove it immediately, I just let it be.

Participant #7

However, not all participants were quite so content with their outcomes. Some mentioned needing further treatment and wanted to have another session with VR. These participants argued that they needed further exercise and more information about how to deal with their fears, referring to several episodes in their daily life when they became overwhelmed and cried when seeing a spider. Continued practice also proved to be difficult for some participants, as a change in seasons from summer to winter made it impossible to find spiders where they lived. One participant was also skeptical about the dissemination of VRET to a wider audience, highlighting the fact that the hefty price tag of an HMD would deter a lot of people from using this type of treatment by themselves.

But I cry and scream, oh man, it’s so bad, it’s really awful to feel this way. Going up in the middle of the night, I have to turn on the lights everywhere so that I don’t miss a spider. It’s difficult, and you also feel stupid, because I know that they’re not dangerous.

Participant #2

## Discussion

### Principal Findings

To our knowledge, this is the first study to use qualitative methods to examine user experiences of undergoing automated, gamified VRET. Using inductive thematic analysis, we recovered several extant topics and open research questions in the field of clinical VR research, and also uncovered novel themes that may guide future quantitative research into the design of VRET interventions and the moderators and mediators of successful outcomes.

Several distilled themes, subthemes, and codes were related directly or indirectly to the sense of presence in the virtual environment [33]. Overall, the app appears to have evoked a strong sense of presence, in some cases to the surprise of the user. Meta-analytic research has confirmed a robust but relatively weak association between self-rated distress and sense of presence during exposure [34]; however, the precise nature of the association, and in particular the direction of causality [35], remains poorly understood. A smaller, single-subject replication trial on the same VRET intervention featured in the current study found no correlation between intervention outcomes and presence rating [20]. Presence is a potential moderator or mediator of intervention effects in VR for mental health. In VR relaxation [21] and VR pain management [36], presence is likely a mediator of intervention effects due to the inherent correlation with distraction from the outside and inside world, the mechanism through which the intervention is hypothesized to work. In VRET, presence is likely a moderator of intervention effects: an adequate sense of presence could arguably be considered a prerequisite for evoking a fear response that can then be attenuated through the same mechanisms as in traditional exposure therapy [37]. Congruently, reported correlation coefficients between presence and pain are typically of stronger magnitude than those reported between presence and distress during VRET [34,38,39]. However, distress in itself has been found to increase presence in VRET [40]. More psychometric research is needed to better separate distress and presence during VR exposure as two separate constructs, which would facilitate in-depth experimental research on how sense of presence moderates experiences during exposure and subsequent outcomes. Using behavioral measures of presence [41] in future research, as opposed to the typical self-reports, may also help to resolve this elusive question.

Further related to the issue of presence, several participants mentioned how realistically the spider locomotion was recreated in the app and how this increased distress. Locomotion-related aspects do indeed appear to be the primary fear-inducing characteristics of spiders [42], revealing the importance of carefully surveying and capturing the fear-relevant aspects of phobic stimuli when developing VRET interventions in order to evoke a strong fear response and sense of presence. Further, several distilled themes and codes concerned threats to presence. Replicating prior qualitative research on VRET [22], technical problems emerged as a prominent theme. In the subsequent replication trial, participants reported comparatively few instances of severe technical issues (sample average app restarts 0.3, SD 0.56, due to overheating for example) [20], although instances of minor technical issues remain unknown. Research on this aspect is difficult due to individual differences in thresholds: even though technical issues could be detected and logged automatically, such issues may be perceived very differently by different users. Some of the issues raised in the current study are generic to the VR field (eg, glitches, bugs, and other software issues), whereas others appeared to be specific to the mobile platform type or even the specific device model used. Compared to tethered VR platforms, mobile VR devices are computationally limited, resulting in lower graphical quality (eg, codes “lack of detail” and “not real enough”). Mobile VR devices also require recharging and are significantly more prone to overheating. It should be noted that mobile VR devices that can accommodate glasses, have higher-quality graphics, and with better battery and heat dissemination capacities have been released since the time of data collection for the current study. Thus, the presentation of this theme and subtheme will likely change over time.

Technical problems are also indirectly related to sense of presence, which in VRET research has typically been measured as a numeric construct at specific time points, with the implicit assumption that the underlying experience can be adequately captured by a measure averaged across some duration. However, it is also possible that measurements at a higher temporal resolution would reveal considerable fluctuations within a distressing task, perhaps even appearing as a near-binary variable since presence is easy to break rapidly, combined with a rapid (new) fear response that is evoked by even minor variations in engagement or stimulus behavior (eg, a still spider suddenly moving). By contrast, technical problems such as overheating and obvious glitches will likely immediately break presence and two occurrences can be sufficient to condition the user to expect more, thereby also attenuating presence in between occurrences. Preventing such issues should be a development priority; however, as evident by the large effect sizes observed despite their occurrence, it need not be catastrophic for outcomes (at least on a group level). Another possible and likely source of rapidly decreasing presence is deliberate safety behaviors. Subtle safety behaviors are common with in vivo exposure therapy [43] and are likely to also occur in VRET, although, to our knowledge, this has not been studied systematically. Deliberately decreasing one’s sense of presence (eg, by focusing on glitches or leaking light) would likely function as a potent, VR-unique safety behavior. Thus, from measurement error alone—distress and presence not being fully disentangled measurements—rapid fluctuations in presence during an exposure task are likely to occur. Future research should attempt to measure presence at a higher temporal resolution, combined with objective measures of technical issues (such as glitches) and safety behaviors (eg, gaze directed away from the phobic stimulus), and statistically model possible causal scenarios.

With respect to VR-unique safety behaviors, participants in the current study mentioned several advantages of VRET, some of which are directly related to what would otherwise be considered an issue of low sense of presence; for example, codes like “feeling safe,” “easier than the real thing,” and “less afraid” reveal that a subset of users were not fully immersed, but that this was not necessarily detrimental to the therapeutic process. Recent empirical research has challenged the long-held assumption within the exposure therapy field that safety behaviors are always detrimental to treatment [44,45]. By inherent design, gamified VRET may be an appealing compromise that offers the user full control in what is expected to be a less distressing experience. Indeed, some research suggests that individuals with phobias show a preference for VRET over in vivo exposure therapy when given a choice [46,47], although it is uncertain to what degree this functions as an avoidance behavior. Future research should examine the role of pretreatment preferences and the occurrence of VR-specific safety behaviors, along with the correlations with outcomes.

As another aspect related to threats to presence, participants in the current study discussed the lack of tactile stimulation (exposure) as a source of skepticism toward the treatment. In an in vivo exposure scenario, touching and holding a spider are among the final steps of treatment [48], a therapeutic ingredient that could not be mimicked in the VRET intervention examined (limited by the consumer technology available at the time), which could partially explain the superiority of in vivo exposure in the clinical trial [19]. Although early research suggested that tactile augmentation of VRET increases the sense of presence, perceived realism, and led to better treatment outcomes [49], subsequent research has failed to replicate these findings [50], leaving an open question to be examined in future research. Of note, since all modern VR platforms feature hand controllers, or even camera-based mapping of actual hand movements, it is now possible to have users experience having virtual hands synchronized to actual hand movements. Future research should examine whether relying on phenomena akin to the Rubber Hand Illusion [51] (eg, seeing a virtual spider crawling up one’s virtual arm) has similar augmenting effects as tactile stimulation (if any) while being logistically easier to deploy until consumer VR hardware platforms integrate tactile stimulation.

Surprisingly, the specific lack of a real-life therapist did not emerge as a theme or even subtheme, and few individual codes were directly associated with the virtual therapist, indicating that the automated format per se did not stand out as a prominent topic. Although we cannot rule out the possibility that this was due to the pilot nature of the study (no study on automated VRET had been published at the time), an inadequate level of detail in the semistructured interview guide (which did however explicitly cover the virtual therapist), or interviewer decisions during the interview, another plausible interpretation is that the automated format was simply perceived as natural and that the gamification elements were successful in framing the experience not as psychotherapy devoid of a therapist but rather as a serious game with a psychotherapeutic goal. Codes such as “forgetting it is treatment,” “well done,” “pedagogical,” “tasks,” and “increasingly more difficult” indicate that the intervention succeeded in blending classic exposure therapy elements (such as psychoeducation and progression along a fear hierarchy) with gamified elements. These qualitative findings thus complement previous quantitative research revealing the high efficacy of automated VRET [17-19] in showing that these interventions are also perceived as appealing. Interestingly, all three studies on automated VRET [17-19] have included a virtual therapist in some format. Recent research has shown that users can develop a relationship similar to a working alliance with either the VR intervention itself [52] or a VR therapist [53]. Participants in this study reported appreciating the calming voice of the virtual therapist, and indicated that it helped them understand their fears better. However, it remains unknown whether the alliance to a virtual therapist has a direct causal role or simply functions as a reminder of the therapeutic context of the serious game. Although initial research has revealed a correlation between alliance and outcomes [53], future randomized controlled trials must experimentally manipulate the availability and format of the virtual therapist to obtain firm conclusions about the therapist’s causal role in automated, gamified VRET outcomes.

### Strengths and Limitations

The current study has both methodological strengths and limitations that should be recognized. First, participants responded to open-ended questions during an interview that was conducted by someone not directly involved in the provision of their treatment. This may have helped them to express their opinions more freely, while also providing more detailed and vivid information than relying solely on survey questions. Nevertheless, given that participants were recruited from those seeking participation in a clinical trial, some participants might have been reluctant to provide more negative views of their experiences due to social desirability effects. Second, providing a transparent and step-by-step description of the analytic process increases the credibility of the results; that is, to what extent any conclusions and inferences made from the interview material seem trustworthy and whether the procedures involved can be replicated. However, the current study did not include a second, independent coding of the material, entailing that the reliability of the uncovered themes is unknown. Third, this pilot study featured a relatively small sample size of 7 participants, which, although justified conceptually and in line with minimum sample sizes in the greater extant literature [26], imposes limitations as to interpretation of the findings. Influential empirical research on theme saturation suggests that although theme fundamentals emerge by 6 interviews, saturation occurs after twice that amount [54]. For this reason, we believe that although this pilot qualitative study is a first explorative attempt to map user experiences of VRET resulting in a preliminary set of themes and subthemes, it is not unlikely that other, low-prevalence themes were not uncovered and that the material may not have been thematically saturated. Therefore, future qualitative work should further explore the themes uncovered in a larger and more diverse sample. In addition, some objective measures describing similarity of experiences such as the exact intervention duration, progress (ie, levels completed), experienced difficulty, and interactions with the technician were not systematically recorded in the current study; however, the intervention itself and format of delivery were highly standardized, which should increase similarity of experiences. Fourth, the issue of transferability in qualitative research is also an important consideration, referring to the extent to which the findings are applicable to another setting or circumstance [55]. Similar studies on participants undergoing other automated, gamified VRET interventions should be conducted to confirm that the themes that emerged in the current study hold true to the larger field of automated, gamified VRET. This includes experiences related to undergoing VRET in complete solitude [20], both in clinical and home settings. The physical presence of a technician during the automated treatment in the current study may have had an impact on user experiences, although none of the uncovered themes beyond technical difficulties directly alluded to an aspect related to the role of the technician. This of course does not exclude the possibility that novel themes could be uncovered from users undergoing VRET in complete solitude.

### Conclusions

Gamified, automated VRET appears to be perceived as an attractive treatment modality by users, despite the inherent distressing nature of exposure therapy. The gamification elements appear to have been successful in framing the experience not as psychotherapy devoid of a therapist but rather as a serious game with a psychotherapeutic goal. A high sense of presence, as well as threats thereto, were discussed as both beneficial and detrimental to usage. Future quantitative and qualitative research is needed to further examine these topics and associations with outcomes, as to inform the next generation of automated VRET apps and achieve a positive public mental health impact.

## Abbreviations

BAT: behavioral approach test
FSQ: Fear of Spiders Questionnaire
HMD: head-mounted display
VR: virtual reality
VRET: virtual reality exposure therapy
